# Thermoelectric Inks and Power Factor Tunability in Hybrid Films through All Solution Process

**DOI:** 10.1021/acsami.1c24392

**Published:** 2022-04-22

**Authors:** José F. Serrano-Claumarchirant, Bejan Hamawandi, Adem B. Ergül, Andrés Cantarero, Clara M. Gómez, Pankaj Priyadarshi, Neophytos Neophytou, Muhammet S. Toprak

**Affiliations:** ^†^Institute of Materials Science (ICMUV), ^‡^Institute of Molecular Science (ICMol), University of Valencia, 46980 Paterna, Spain; § Department of Applied Physics, KTH Royal Institute of Technology, SE-106 91 Stockholm, Sweden; ∥ School of Engineering, University of Warwick, Coventry CV4 7AL, U.K.

**Keywords:** thermoelectric, organic−inorganic hybrids, thermoelectric power factor, interface engineering, nanoparticles, bismuth telluride, antimony telluride, microwave synthesis

## Abstract

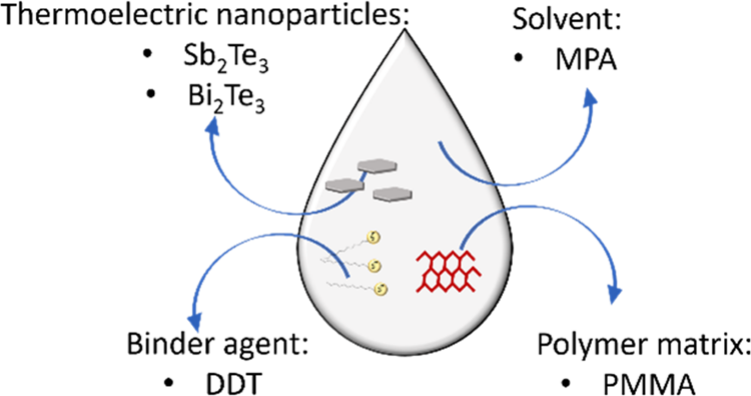

Thermoelectric (TE) materials can have a strong benefit to harvest thermal energy if they can be applied to large areas without losing their performance over time. One way of achieving large-area films is through hybrid materials, where a blend of TE materials with polymers can be applied as coating. Here, we present the development of all solution-processed TE ink and hybrid films with varying contents of TE Sb_2_Te_3_ and Bi_2_Te_3_ nanomaterials, along with their characterization. Using (1-methoxy-2-propyl) acetate (MPA) as the solvent and poly (methyl methacrylate) as the durable polymer, large-area homogeneous hybrid TE films have been fabricated. The conductivity and TE power factor improve with nanoparticle volume fraction, peaking around 60–70% solid material fill factor. For larger fill factors, the conductivity drops, possibly because of an increase in the interface resistance through interface defects and reduced connectivity between the platelets in the medium. The use of dodecanethiol (DDT) as an additive in the ink formulation enabled an improvement in the electrical conductivity through modification of interfaces and the compactness of the resultant films, leading to a 4–5 times increase in the power factor for both p- and n-type hybrid TE films, respectively. The observed trends were captured by combining percolation theory with analytical resistive theory, with the above assumption of increasing interface resistance and connectivity with polymer volume reduction. The results obtained on these hybrid films open a new low-cost route to produce and implement TE coatings on a large scale, which can be ideal for driving flexible, large-area energy scavenging technologies such as personal medical devices and the IoT.

## Introduction

Thermoelectric (TE) materials can interconvert between heat and electricity, and this ability has not only led to remarkable technologies such as power generators used in space exploration but also holds an obvious potential in the green transition to recover waste heat. The efficiency of TE energy conversion increases with an increasing figure of merit −ZT, defined as *S*^2^σ*T*/κ, where *S* is the Seebeck coefficient (=−Δ*V*/Δ*T*, the voltage (−Δ*V*) induced by a temperature gradient (Δ*T*)), σ is the electrical conductivity, κ is the thermal conductivity, and *T* is the absolute temperature. It is an exciting scientific challenge to find materials that optimize the strongly negatively correlated material properties such as *S*, σ, and κ. Nanostructuring has become an important approach in the field of TEs for reducing the thermal conductivity κ independently from the electrical conductivity σ.^1−5^ For the widespread application of TE materials, it is equally important that the structural integrity and chemical stability of the materials are preserved under operating conditions.

The most common TE material for room-temperature operations up to about 150 °C is Bi_2–*x*_Sb*_x_*Te_3_ alloys, where the type of conduction is n-type for Bi-rich compositions and shifts to p-type for Sb-rich compositions. This material is heavily studied using a variety of solid-state and solution chemical synthetic techniques. Bottom-up chemical methods are commonly used because of their low cost, low investment need, and the possibility of scale-up of the synthesis process. Different morphologies of Bi_2–*x*_Sb*_x_*Te_3_ including nanoparticles, nanoplates, nanocrystalline films, nanorods, nanotubes, nanowires, and nanoflowers have been synthesized by reverse micelles, metal-organic chemical vapor deposition, vapor–liquid–solid, refluxing, electrodeposition, chemical reduction, solvothermal, hydrothermal, ultrasonic-assisted, and microwave (MW)-assisted routes.^1,5,6^

On the other hand, there is a significant amount of work focusing on hybrid TE materials, where solid TE materials in the form of compacted pellets, porous media, nanowires, or nanoparticles are integrated within organic species and inks to create flexible TEs or to alter the properties of either the solid-state or organic parts.^7−9^ These materials typically have significantly reduced performance and ZT compared to their purely solid-state counterparts; however, they have the advantage of being flexible, cost-effective, and typically target room-temperature microscavenging type of applications related to IoT and powering flexible electronics, for which harvesting enough, rather than too much energy, is required. An important element of these materials is the identification of directions for improvement in the performance of the solid-state material upon the interaction with the organic part.

In this work, we developed Sb_2_Te_3_ and Bi_2_Te_3_ nanoparticles synthesized through MW-assisted route and used them to explore the possibility of formulating TE inks. The synthesized Sb_2_Te_3_ and Bi_2_Te_3_ nanoparticles were embedded in poly(methyl methacrylate) (PMMA) at various weight fractions. Thick films in the range of 1–5 μm were fabricated by spin-coating using (1-methoxy-2-propyl) acetate (MPA) as the solvent. Furthermore, performance boosting, as a result of the incorporation of 1-dodecanethiol (DDT), of the TE transport properties of the hybrid films is demonstrated. Contrary to the common view, the addition of DDT increases (rather than decreases) the electrical conductivity, possibly as a result of the creation of more compact structures, which would increase the overall surface overlap of the platelets (and thus reducing the interparticle resistance) and/or creating better percolation networks. The long-term stability of the hybrid films stored in air for prolonged periods is studied, revealing the protector role of PMMA on the Sb_2_Te_3_ and Bi_2_Te_3_ nanoparticles.

## Results and Discussion

**Figure 1:**
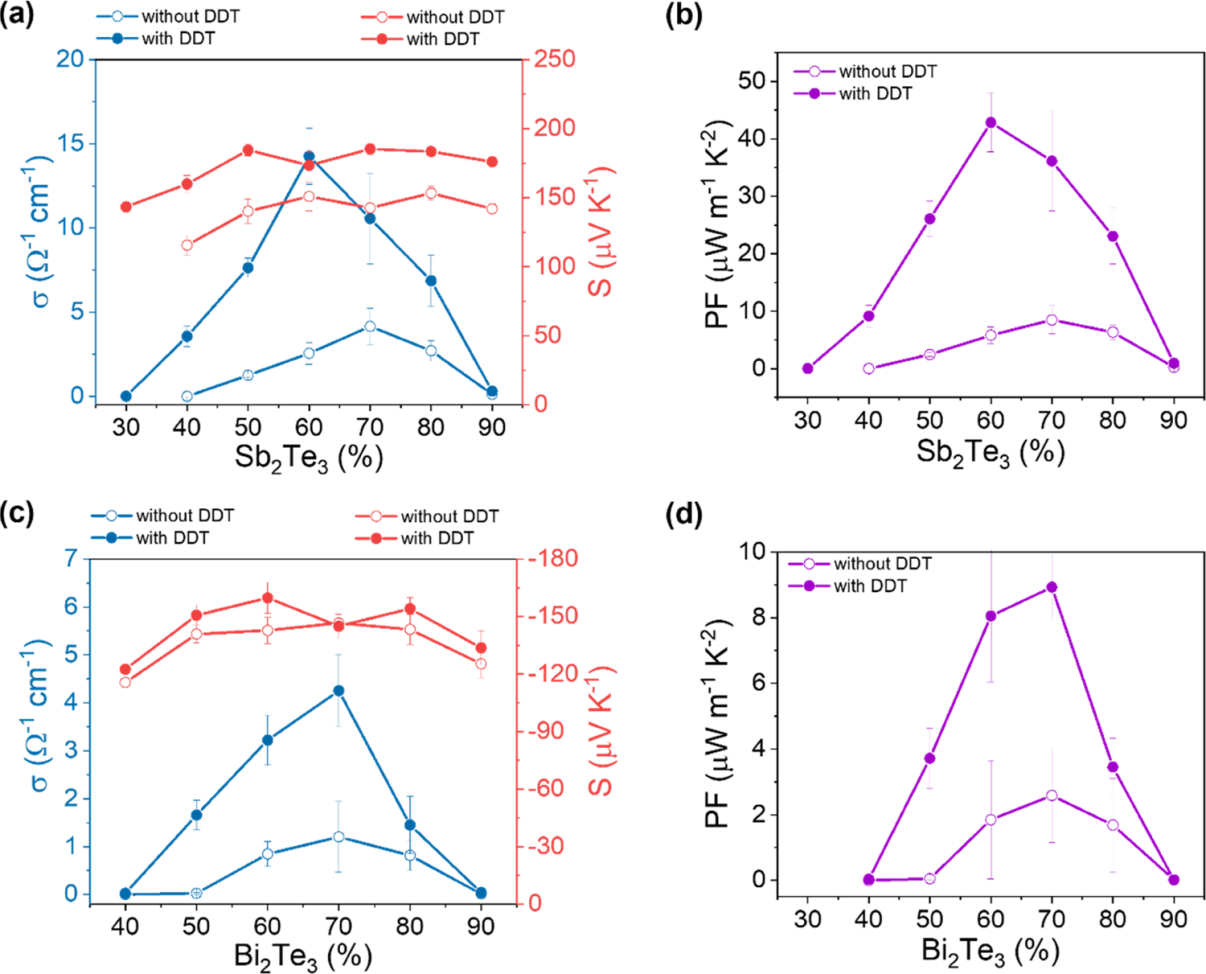
Electronic transport properties of the hybrid films with varying contents of nanoparticles in the absence and presence of DDT; (a) electrical conductivity (σ) and Seebeck coefficient (*S*), (b) power factor (PF) for Sb_2_Te_3_ hybrid films; (c) σ and *S*, and (d) PF for Bi_2_Te_3_ hybrid films.

**Figure 2:**
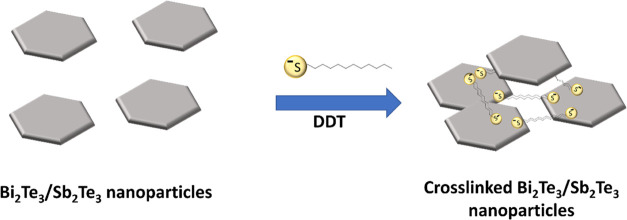
Proposed mechanism (not to scale) of improved interconnectivity of Sb_2_Te_3_ and Bi_2_Te_3_ nanoparticles in the hybrid films by the addition of DDT.

Phase purity of the as-made materials has been studied using XRD and the diffraction patterns showing a high phase purity for Sb_2_Te_3_ and Bi_2_Te_3_, as presented in Figure S1. These materials were then used to formulate the TE inks and the hybrid films in the PMMA matrix. The **σ** of the hybrid Sb_2_Te_3_ films (Figure 1a) gradually increases with solid content until reaching a content between 60 and 70%. From this point on, however, surprisingly the electrical conductivity drops despite the increasing solid content. We believe that the reason for this is that the particles are no longer fully embedded in the PMMA polymer matrix as their content increases substantially, leading to conductivity-reducing microstructural differences in the film formation. On the other hand, the *S* (red lines) of the hybrid Sb_2_Te_3_ films does not seem to change much with the variation of the solid content, retaining values of around 150–175 μV K^–1^. Interestingly, however, we find that σ can substantially increase with the addition of DDT, which improves the connectivity of the conductive network (schematically shown in Figure 2). In addition, DDT also gives better rheological properties to the ink formulation due to the increase of viscoelasticity.^10^ It triples the electrical conductivity of the original film, accompanied also by a slight increase of the Seebeck coefficient (rather than decrease as one would have expected). Therefore, the power factor (PF) −*S*^2^σ of the hybrid films with DDT increases by five times compared to those without DDT (Figure 1b).

In the case of Bi_2_Te_3_ hybrid films, a similar trend of increasing σ was obtained with solid content, until reaching a content of 70% (Figure 1c). Then, the σ decreases, probably again due to the inhomogeneous distribution of the particles in the PMMA matrix or the reduced thickness of the PMMA on the nanoparticles influencing the connectivity of the films. The *S* does not change with the increasing Bi_2_Te_3_ content, remaining around −150 μV K^–1^ (Figure 1d). With the addition of DDT, the σ increases by three times, while the *S* stays almost the same (only a slight increase is observed), resulting in PF enhancement of four times compared to the PF of hybrid films without DDT (Figure 1d).

**Figure 3:**
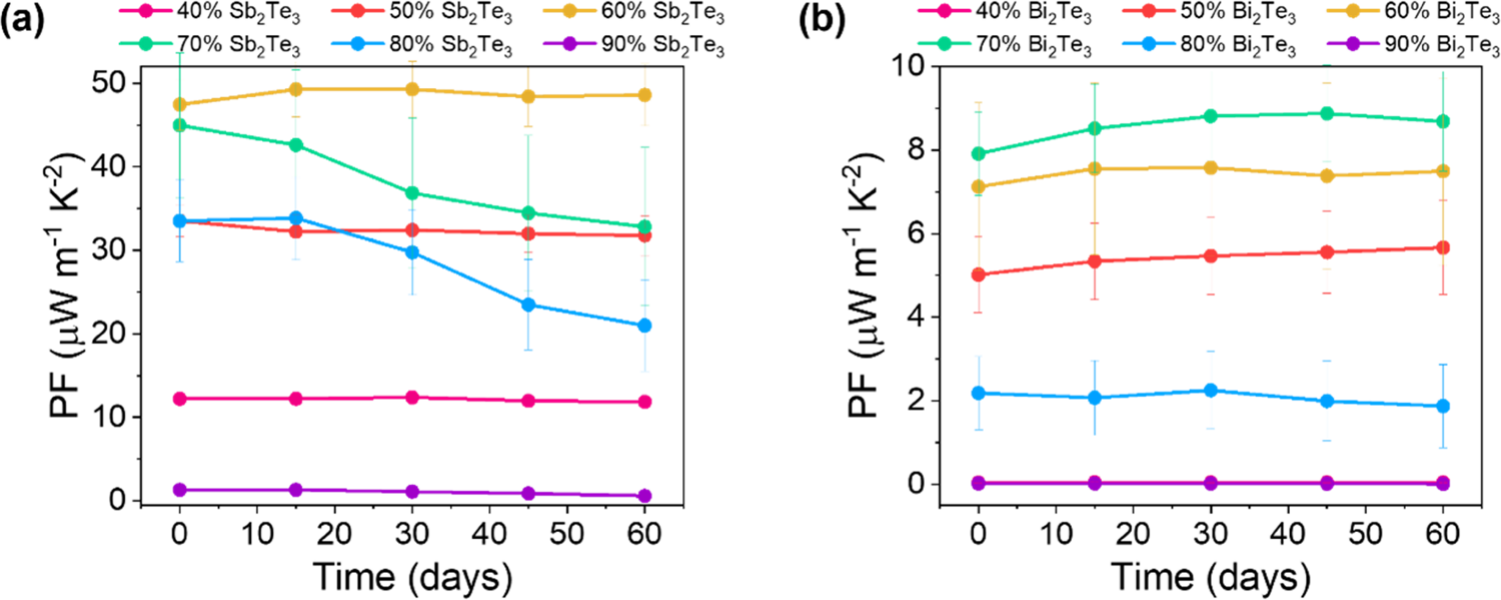
Stability test of the optimized hybrid films based on the PF for (a) PMMA–Sb_2_Te_3_ and (b) PMMA–Bi_2_Te_3_ with the addition of DDT.

**Figure 4:**
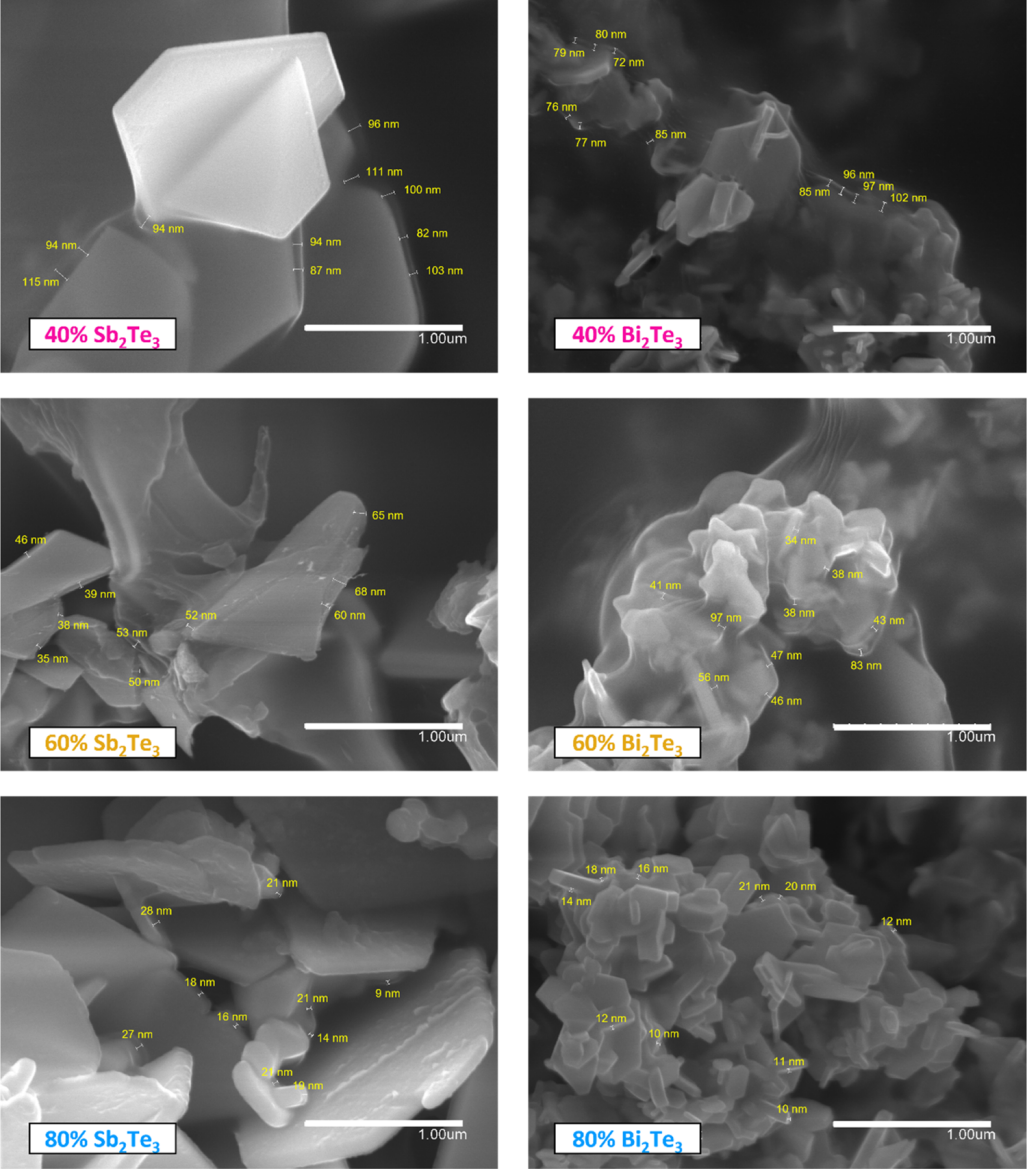
SEM micrographs of some selected hybrid films with different contents of Sb_2_Te_3_ and Bi_2_Te_3_ nanoparticles marked with the PMMA coating thickness.

One of the major concerns of chalcogenide-based TE films is their stability against environmental conditions since these can cause oxidation of the materials and, therefore, a decrease in their performance over time.^11−15^ PMMA is a stable polymer up to 120 °C; it is hydrophobic and impermeable to air.^16−19^ Embedding Sb_2_Te_3_ and Bi_2_Te_3_ nanoparticles in a polymeric matrix of PMMA can allow the final film to stay protected against environmental conditions. The stability of the optimized films with DDT has been studied as a function of time under ambient conditions at 25 °C and 67% humidity. The PF of the films remained rather stable for most of the sample compositions after two months from their deposition, as can be observed in Figure 3a,b. In the case of Sb_2_Te_3_ films, the PF remains stable up to a solid content of 60%. At higher percentages (70–80%), it decreases progressively, probably due to the thinner polymeric coating and surface oxidation of Sb_2_Te_3_ nanoparticles with large lateral dimensions. The difference in the PF stability of hybrid Bi_2_Te_3_ films at high solid content, as compared to that of Sb_2_Te_3_ films, is attributed to the smaller size of Bi_2_Te_3_ nanoparticles, resulting in a more effective coating with the polymer than the larger Sb_2_Te_3_ nanoparticles. In addition, it can be observed from the SEM micrographs in Figure 4 that as the solid content in the hybrid film increases, the PMMA coating on the Sb_2_Te_3_ and Bi_2_Te_3_ nanoparticles gets thinner, decreasing from about 100 nm (in 40% solid content) to about 15 nm (80% solid content) (some additional cross-sectional SEM micrographs of these films are presented in Figure S2). The decrease in the thickness of the PMMA coating on the nanoparticles helps to explain the observed stability reduction with increasing solid content, since a lower coating thickness implies weak protection of nanoparticles against environmental humidity and oxidation.

### Transport Description and Modeling

#### Electrical Conductivity

**Figure 5:**
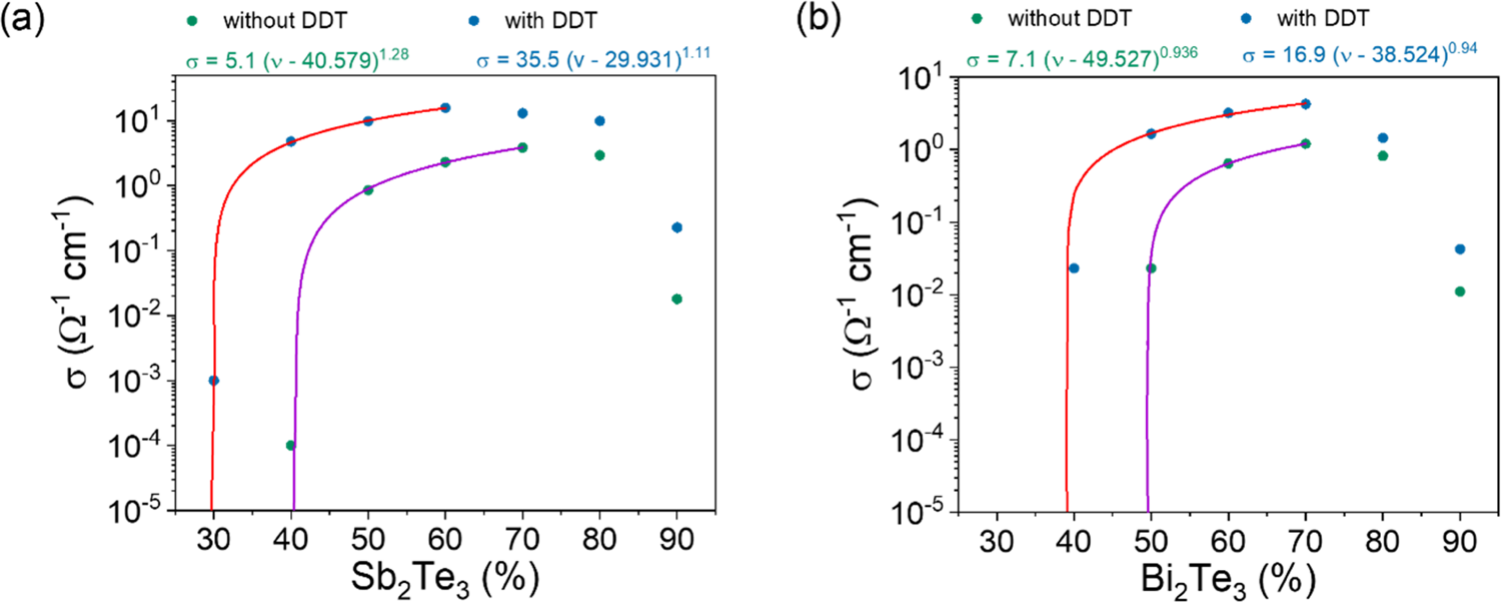
Electrical conductivity and percolation curves for (a) Sb_2_Te_3_–PMMA and (b) Bi_2_Te_3_–PMMA hybrid films. Percolation fitting with the fitting parameters is specified above each graph for hybrid samples with and without the addition of DDT.

For the σ, for the low solid densities, we observe percolation behavior, with the general trend involving the percolation threshold volume and the percolation exponent, as^20^
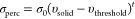
1The σ as a function of the solid content of Sb_2_Te_3_ and Bi_2_Te_3_ shows a percolation behavior up to 60% content and before the conductivity takes the downturn (Figure 5a,b, respectively). To determine the percolation threshold, experimental data were fitted to eq 1 and, in both the cases, the addition of the linker DDT reduces the percolation threshold without noticeably varying the critical exponent. When the percolation threshold of Sb_2_Te_3_ and Bi_2_Te_3_ hybrid films with the DDT linker is compared, the value obtained for Sb_2_Te_3_ is lower than that of Bi_2_Te_3_. As the percolation threshold depends on the particle size, the results suggest that the Bi_2_Te_3_ nanoparticles are smaller than the Sb_2_Te_3_. This can be easily confirmed from the SEM micrographs of as-made Sb_2_Te_3_ and Bi_2_Te_3_ nanoparticles presented in Figure S3 (see the Supporting Information for further discussion). Furthermore, from fitting the experimental results, the value of the critical exponent *t* is estimated around 1, which is the predicted value for 2D conductive networks from the classical percolation theory.^20,21^

To create a more comprehensive model of the measured behavior, we assume the following process: the fact that percolation transport is observed at high polymer densities (low solid material densities) is a strong indication that transport is through the solid material primarily, effective only in the small number of transmission paths that form, and the polymer remains relatively nonconductive (or less conductive). Otherwise, we would have observed higher **σ** at higher polymer densities. At moderate and high solid fraction/volume, transport is assumed to gradually become effective medium-like, where charge flows in the solid material platelet and encounters interface resistances between the platelets. We cannot ignore the fact that boundary and defect scattering will be present within the platelets due to their possible polycrystalline nature. Therefore, we assume mild scattering only internally in the platelets. We ignore transport in the polymer whose volume is being reduced anyway. We assume, however, that the polymer facilitates the connectivity and interface resistance between the platelets. Thus, the change of the polymer nature as its volume decreases increases the interface resistance significantly as observed in the measurements and gradually diminishes the overall conductivity.

Assuming a general analytical series resistance model for the large solid volume fraction regime, which accounts for the intrinsic resistance of the solid materials, potential defects, and grain boundaries, and the interface resistance between the connecting platelets, the overall electrical conductivity is given by^22,23^
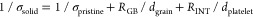
2where *R*_GB_ is a parameter defining the resistance of the internal grain boundary interface (with units Ωm^2^, such that *R*/*d* provides resistivity), *d*_grain_ is the distance between internal grain boundaries, *R*_INT_ is the interface resistance between platelets, and *d*_platelet_ is the effective distance between platelets. Note that the addition of DDT linkers can in this way be accounted by reducing *R*_INT_. We also make the arbitrary assumption that  to indicate that the platelet interface resistance increases as the polymer volume (*v*) is reduced—*R*_0_ is considered to be an arbitrary constant to fit the data (essentially the only fitting parameter we use in the model). Using  versus  does not capture as steep a reduction in conductivity as observed for large solid volumes, which makes us conclude that the interface resistance increases disproportionally with polymer volume reduction. This larger than linear increase in the interface resistance is needed to obtain the qualitative trend of the measured data. Note that the properties of these interfaces and the way they facilitate transport and connectivity are largely unknown, and we were not able to extract those from the measured data in this work at the moment. Transport effects from purely resistive, hopping, and even tunneling in the cases where the regions are thinner than a few nanometers (as the polymer volume decreases) could all take part. In the absence of specific details, we lump all of these effects into a comprehensive value for the interface resistance, which is determined by fitting to the measured data. For example, a smaller distance between the nanoparticles could potentially improve tunneling and hopping and lead to better connectivity, which essentially translates to lower interface resistance. The same with increasing the overlapping surface in more compact structures resulted after the addition of DDT, which would also increase connectivity.

We finally combine the conductivities of the percolation and the series resistance model by weighing them with the volume fraction of the polymer versus the solid materials as
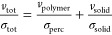
3

#### Seebeck Coefficient, ***S***

The *S* is at first order the distance of the band edges from the Fermi level as
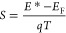
4This does not change with solid volume significantly since the carrier density does not change. Essentially, the assumption is that as the solid volume increases, more conducting paths are added, but all are of the same nature, giving the same *S*. The measured *S* values for both Sb_2_Te_3_ and Bi_2_Te_3_ are very close to the literature values for these materials (Winkler et al. reported that *S* for Sb_2_Te_3_ is between *S* =130 and 150 μV K^–1^,^24^ very close to our measured values), which again indicates that transport is through the solid material and the polymer does not contribute to transport or to *S*.

However, since scattering off interfaces increases as the polymer volume decreases and its connecting ability decreases, we would have expected an increase in the *S* by potential energy filtering effects that might take place. The effect is small, however, indicating that the overall interface volume fraction is small compared to the solid material volume, and/or only small potential barriers are formed, such that the *S* increases only by 10–20%.

For the hybrid material, we model the overall ***S*** similarly to the conductivity, i.e., as a series combination of crystalline solid, with grain boundaries (in the solid), and polymer interface parts between the platelets, with weighing factor the volumes of the three regions
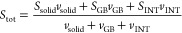
5Since *S* varies only a little as observed from the experiment, the variation above originates mostly from the platelet interface, rather than the grain boundaries internal to the platelets. Here, we also make an assumption about *v*_INT_, as *v*_INT_ = *v*_solid_^2^. This seems rather arbitrary, but it essentially resembles an interface surface region surrounding the platelets and can capture the slightly increasing trend of the *S* with solid volume increase (since these are volume fractions, it holds that v_INT_ < v_solid_ as expected). Above, values for the *S* of the solid material, *S*_solid_, can be found in the literature. *S*_INT_, on the other hand, is not known, and it is used as a parameter to fit the measured data (essentially taken from the measured data).

Overall, some parameters needed for the models are found in different publications (although variations exist), including our previous work, but some others are not easy to identify.^3,24^ Thus, we make reasonable choices according to structural characterization data to calibrate the models to map to the measured data.

#### Parameters and Results for Sb_2_Te_3_–PMMA Hybrid Films

**Figure 6:**
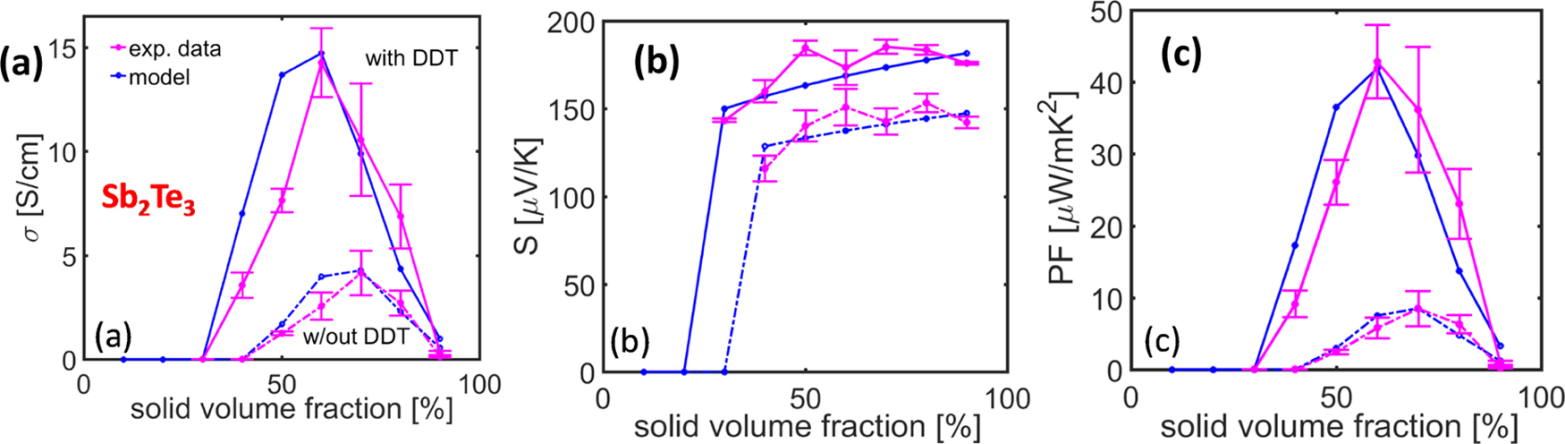
Sb_2_Te_3_–PMMA model versus measured values for the (a) σ, (b) *S*, and (c) PF. Results w/out and with the addition of DDT are shown.

First, for the case of Sb_2_Te_3_, for low solid densities, we have identified that the percolation treatment σ_perc_ = 5.1(υ_solid_ – 40.479)^1.28^ provides an excellent match to the measured data (see Figure 6a). Then, we use σ_pristine_ = 2300 S/cm, which agrees well with the literature.^3^ We assume that the intrinsic phonon-limited mean free path is λ_solid_ = 100 nm.^25^ From the structural characterization, we obtained platelets with a crystallite size in the range of 160–600 nm, with an average crystallite size of about 200 nm (for more details, see the Supporting Information). Thus, we assume that such structural internal grain boundary scattering would contribute to an additional scattering similar to that of phonons, such that 1/σ_pristine_ = *R*_GB_/*d*_grain_ in the conductivity (eq 2). For *d*_grain_ = 200 nm, then *R*_GB_ = 8.7 × 10^–13^ Ωm^2^. With regard to the size of the platelets, from structural characterization, we find that the Sb_2_Te_3_ average lateral particle/platelet size is 1.5 μm (with a std. dev. of 0.7 μm). We then adjust the value *R*_0_ = 3 × 10^–10^ Ωm^2^ to obtain the best match with the measured data. Note that the  with the chosen value of the platelet connection resistance of *R*_0_ is the only “strong” assumption we make in this analysis.

For the Seebeck coefficient, we assume that a typical grain boundary thickness internally in the material is *d*_GB_ = 2 nm, which results in the grain boundary volume being approximately 1% of the crystallite volume. Using *S*_solid_ = 100 μ*V*/*K* and *S*_defect_ = 200 μ*V*/*K* provides a good match to the measured data. The power factor finally is also matched adequately. Note that in the absence of knowledge about the *S*_GB_ values, and due to the fact that internal grain boundaries occupy minimal space and are also expected to affect the overall performance minimally, we arbitrarily pick *S*_GB_ to be the middle value of *S*_solid_ and *S*_defect_, as *S*_GB_ = 150 μ*V*/*K*. Overall, due to the high crystallinity of the crystallites, the effect of the internal grain boundaries on the overall *S* turns out to be negligible, but we include it in the model for consistency.

#### Addition of DDT in Sb_2_Te_3_–PMMA Hybrid Films

In the case of the addition of DDT in the Sb_2_Te_3_–PMMA hybrid structure, the percolation equation that describes the data for low solid densities is σ_perc_ = 35.5(υ_solid_ – 29.931)^1.1^. The addition of linkers can be modeled by only reducing *R*_0_ in the conductivity (eq 2). The measured data can be matched very well if we set *R*_0_ = 1.67 × 10^–7^ Ωm^2^, as observed in Figure 6a (almost half of the value used without DDT linkers). This could indicate that the use of linkers created a denser structure (see Figure 2), and thus the lower percolation threshold and that the resistance between platelets are reduced by a factor of almost 2 (with the rest of the conductivity increase attributed to the denser network).

For the Seebeck coefficient, using *S*_solid_ = 120 K^–1^ and *S*_defect_ = 250 μV K^–1^ provides a good match to the measured data. The power factor finally is also matched adequately, indicating a 5-fold improvement compared to the structure without linkers (Figure 6c).

#### Parameters and Results for Bi_2_Te_3_–PMMA Hybrid Films

For the case of Bi_2_Te_3_–PMMA hybrids, for low solid densities, we have identified that the percolation relation σ_perc_ = 7.1(υ_solid_ – 40.527)^0.936^ provides an excellent match to the measured data. Then, we use σ_pristine_ = 1000 S cm^–1^, which agrees well with the literature.^3^ We make the assumption that the intrinsic phonon-limited mean free path is also λ_solid_ = 100 nm.^25^ From the structural characterization, we obtained platelets with a crystallite size in the range of 50–100 nm with an average crystallite size of 70 nm. For simplicity, we also assume that such structural internal grain boundary scattering would contribute to an additional scattering similar to that of phonons, such that 1/σ_pristine_ = *R*_GB_/*d*_grain_ in the conductivity (eq 2). For *d*_grain_ = 70 nm, then *R*_GB_ = 7 × 10^–13^ Ωm^2^. With regard to the size of the platelets, from structural characterization, we find that the Bi_2_Te_3_ average particle size is 200 nm (with a std. dev. of 95 nm). We then adjust the value *R*_0_ = 2.4 × 10^–10^ Ωm^2^ to obtain the best match with the measured data. This value is similar to that used for Sb_2_Te_3_ above (3 × 10^–10^ Ωm^2^), indicating that the solid/polymer/solid interfaces in both cases have similar resistivity.

**Figure 7:**
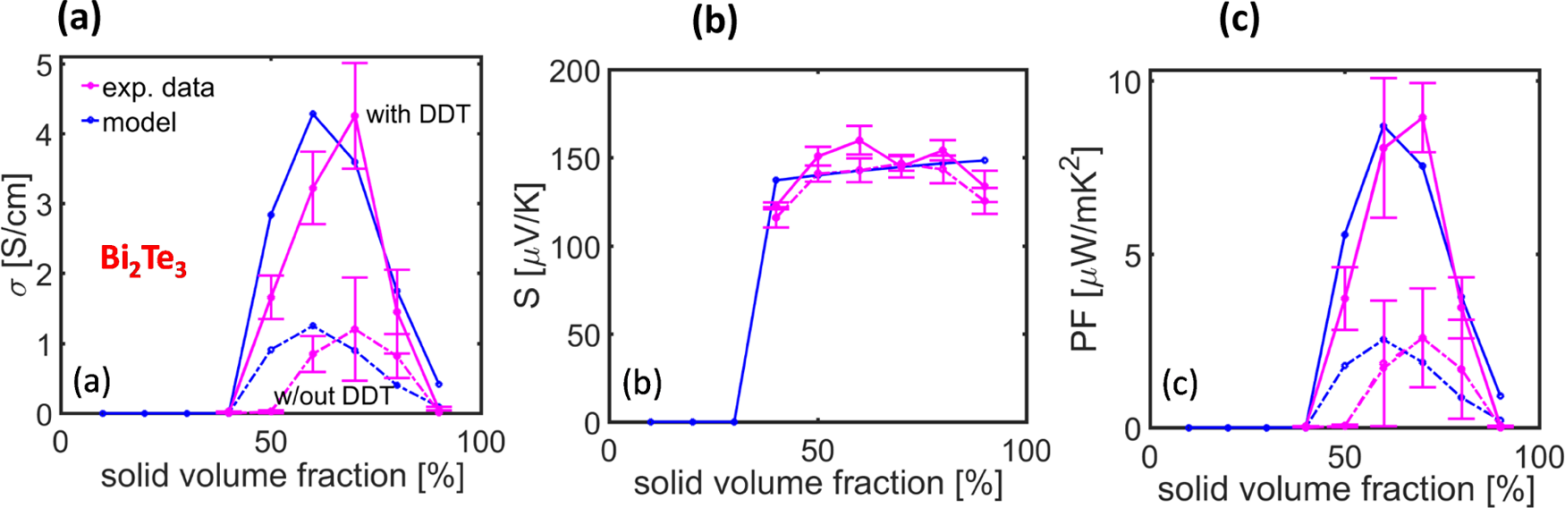
Bi_2_Te_3_–PMMA model versus measured values for the (a) σ, (b) *S*, and (c) PF. Results w/out and with the addition of DDT are shown.

For the Seebeck coefficient, using *S*_solid_ = 120 μV K^–1^ and *S*_defect_ = 180 K^–1^ provides a good match to the measured data. The PF finally is also matched adequately (see Figure 7). In the same way as for Sb_2_Te_3_ earlier, we assume a typical internal grain boundary thickness of *d*_GB_ = 2 nm, which results in the grain boundary volume being approximately 3% of the crystallite volume, and *S*_GB_ = 150 μ*V*/*K*. Here again, the effect of the internal grain boundaries on the overall *S* is negligible.

#### Addition of DDT in Bi_2_Te_3_–PMMA Hybrid Films

In the case of the addition of DDT in the Bi_2_Te_3_–PMMA hybrid structure, the percolation equation that describes the data at low solid densities is σ_perc_ = 16.9(υ_solid_–38.524)^0.94^. The measured data can be matched very well if we set *R*_0_ = 5.33 × 10^–11^ Ωm^2^, as observed in Figure 7a (almost five times lower compared to the value used without DDT linkers). In this case, the use of linkers does not seem to alter the percolation threshold, possibly not creating a denser structure as in the case of Sb_2_Te_3_ above; thus, the change in transport is entirely caused by the resistance between platelets being reduced by a factor of almost 4.5.

For the Seebeck coefficient, using the same *S*_solid_ = 120 μV K^–1^ and *S*_defect_ = 180 K^–1^ as in the case without DDT provides a good match to the measured data (Figure 7b). The power factor finally is also matched adequately (Figure 7c).

#### Discussion on the Models

We need to stress that the system under consideration is overly complex and the construction of the model is informed directly by experimental data. We need to stress, however, that the material system under investigation is overly complex (hybrid material, involving different transport regimes and structure changes upon solid volume change), and it is not possible for any existing model or theory that we are aware of, either simple or advanced, to be able to capture this behavior. Still, however, we begin with the simplest models that exist, i.e., the percolation model and the series resistance model. We then gradually modify the series resistance model based on possible intuitive guesses that align with what we observe in the morphology of the material and the peculiar behavior of the measured data. The modifications point toward possible explanations for the transport observations and thus toward useful physical information. For example, in the case of Bi_2_Te_3_, the model suggests that DDT increases the connectivity between platelets by 4.5×, rather than 2× as in the case of Sb_2_Te_3_, something that cannot be trivially extracted by observing the measured data itself.

In addition, the exact reasoning behind the increase in the conductivity behavior with the addition of DDT is not completely identified. As the most possible explanation, as shown in Figure 2, we believe that DDT makes the structure more compact, with the platelets having a larger degree of surface overlap. This leads to better connectivity as follows: for low solid densities, better percolation paths are formed, and at moderate solid volume densities, the larger overlapping surface increases conduction from one nanoparticle to another. Indeed, better percolation behavior is observed with the addition of DDT, with reduced percolation threshold. It is possible that both better percolation and improvement in the interconnectivity between the particles coexist, most possibly due to increasing surface overlaps. Although DDT decreasing the distances between the nanoparticles can also lead to increase in conductivity, we still need to perform more studies to actually verify precisely if such morphological effect takes place.

### Flexibility Assessment

To study the flexibility limits of the developed hybrid films, we performed tests on the Sb_2_Te_3_–PMMA hybrid film with a 60% Sb_2_Te_3_ content and DDT linker. For this, the deposition of the ink has been applied to a flexible PET substrate. Results are presented in Figure S5a, which shows the results of bending the film 3000 times on a 2 cm diameter cylinder and measuring the change in electrical conductivity after every 100 bendings. The conductivity of the film gradually decreases with the number of flexes until reaching a loss in an electrical conductivity of 50% after 3000 flexes. On the other hand, Figure S5b shows the variation of σ as a function of the bending radius. In this case, the σ also decreases as the bending radius decreases, and a loss of electrical conductivity by 70% is reached when the bending radius is as small as 1 cm. These results clearly indicate that the formulated hybrid films based on Sb_2_Te_3_ (and Bi_2_Te_3_) nanoparticles with DDT and in the PMMA matrix are not particularly flexible. However, we must remember that a film composed solely of Sb_2_Te_3_ (or Bi_2_Te_3_) nanoparticles deposited on a flexible substrate such as PET would almost completely lose its conductivity after a few bending cycles since in this case there would be no glue effect of the polymeric matrix. With the choice of other flexible polymers as the matrix, higher flexibility might be achieved, with much less degradation of the transport performance.

## Conclusions

To conclude, in this work, we explored the possibility of developing p- and n-type thermoelectric inks by embedding in-house-synthesized Sb_2_Te_3_ and Bi_2_Te_3_ nanoparticles with hexagonal platelet morphology in the polymeric matrix of PMMA. We show that the addition of a bridging agent such as DDT significantly increases the thermoelectric power factor of the films due to the improvement of the interfaces and interconnectivity of the conductive network in the matrix. The highest power factors obtained were 47.45 μW K^–2^ m^–1^ for p-type hybrid films based on Sb_2_Te_3_ (60% solid content) and 7.91 μW K^–2^ m^–1^ for n-type hybrid Bi_2_Te_3_ films (70% solid content). We show that the performance is improved up to 60% solid content, after which the PMMA coating thickness around the nanoparticles decreases, essentially reducing the connectivity of the network and the power factor. The observed trends were captured by a model that combines percolation theory and analytical resistive transport theory. Furthermore, these films have been found to be highly stable under ambient conditions. These results open a new low-cost way of producing and implementing thermoelectric coatings on a large scale.

## Materials and Methods

### Materials

Bismuth chloride (BiCl_3_, 98% purity), antimony chloride (SbCl_3_, 98% purity), Te powder (99.8% purity), oleic acid (CH_3_(CH_2_)_7_CH=CH(CH_2_)_7_COOH), 1-octadecene (CH_3_(CH_2_)_15_CH=CH_2_, ≥95.0% purity), thioglycolic acid (TGA, HSCH_2_COOH, 98% purity), tributyl phosphate (TBP, (CH_3_(CH_2_)_3_O)3PO, 97%), (1-methoxy-2-propyl) acetate (MPA, CH_3_COOCH(CH_3_)CH_2_OCH_3_, ≥96.0% purity), dodecanethiol (DDT, CH_3_(CH_2_)_11_SH, 98% purity), and poly (methyl methacrylate) (PMMA, [CH_2_C(CH_3_)(CO_2_CH_3_)]*_n_*, average *M*_w_ ∼ 15 kDa) were all received from Sigma-Aldrich (Sweden) and used as-received, without further purification.

### Synthesis of TE Nanomaterials

Sb_2_Te_3_ and Bi_2_Te_3_ nanoparticles were synthesized through the microwave (MW)-assisted thermolysis process, which significantly shortens the reaction time accompanied by a high yield.^3^ The synthesis is performed in oleic acid, resulting in oleophilic nanoparticles that can be easily dispersed in the polymer solution. In a typical synthesis, the starting materials SbCl_3_/BiCl_3_ are mixed with oleic acid, 1-octadecene, and thioglycolic acid. The Te powder is complexed with tributyl phosphate. Then, these two solutions are mixed in a 100 mL MW reactor, which has been heated up to 220 °C (ramp time of 4 min) and maintained there for 2 min, before cooling down to room temperature.

### Formulation of TE Ink and Hybrid Film Fabrication

The TE inks were formulated by mixing the nanoparticles with a solution of PMMA at 20% by weight using MPA as the solvent. The Sb_2_Te_3_ and Bi_2_Te_3_ nanoparticles are primarily mixed with MPA to make a homogeneous blending when mixed with the PMMA solution in MPA. The volume of NPs in MPA was adjusted so that the PMMA–nanoparticle weight ratio matches the desired weight percent of the hybrid films. Upon mixing both the components, the temperature of the mixture was increased to 80 °C and maintained there under constant stirring for 30 min to assure a homogeneous dispersion quality. Once the TE ink was homogenized, a 100 μL aliquot was taken and dispersed on a glass substrate, previously cleaned with isopropanol, using a spin coater at 1800 rpm for 30 s. 1-Dodecanethiol (DDT), equivalent to 1% of the total weight of the film, was added to the TE ink prior to fabrication of hybrid films containing DDT. Upon coating with the TE ink based on the Sb_2_Te_3_/Bi_2_Te_3_–PMMA hybrid films, the solvent (MPA) was removed by heating the substrate to 80 °C for 10 s. This time was enough to ensure a controllable evaporation of the solvent, by which the reconcentration of material on certain parts of the substrate is inhibited.

### Material Characterization

The electronic transport properties of the hybrid films were determined by the measurement of electrical conductivity (σ) and the Seebeck coefficient (*S*). The σ was determined by the Van der Pauw method. The *S* was determined using a homemade system. The details of evaluation of *S* (Figure S6) and σ (Figure S7) are presented in the Supporting Information. The thickness of the films was measured using a profilometer (Profiler KLA-Tencor P15, CA), while the lateral dimension was measured using a caliper. The dependence of the electrical conductivity and the thickness of the hybrid films based on the number of deposition steps are presented in Figure S8. Morphology and microstructure analyses of the materials were performed using scanning electron microscopy (SEM) (FEI Nova 200, FEI Company, Hillsboro, OR).

## Supporting Information Available

Additional information on the characterization of Sb_2_Te_3_ and Bi_2_Te_3_ nanoparticles, microstructure analysis, flexibility testing, and the details of electrical characterization techniques (PDF)
