# Supplementary material for: Thermoelectric Inks and Power Factor Tunability in Hybrid Films through All Solution Process
Source: ACS Appl Mater Interfaces. 2022 Apr 22;14(17):19295–303. doi: 10.1021/acsami.1c24392 (PMC9073925; doi:10.1021/acsami.1c24392)
Supplement: Supplementary file 1 — am1c24392_si_001.pdf [file am1c24392_si_001.pdf]

Supporting Information

Thermoelectric Inks and Power Factor Tunability in Hybrid Films through All Solution Process

José F. Serrano-Claumarchirant[†], *Bejan Hamawandi*[‡]*, *Adem B. Ergül*[‡], *Andrés Cantarero*[§], *Clara M. Gómez*[†], *Pankaj Priyadarshi*[¥], *Neophytos Neophytou*[¥], *and Muhammet S. Toprak*[‡]*

[†] Institute of Materials Science (ICMUV), University of Valencia, 46980 Paterna, Spain

[‡] Department of Applied Physics, KTH Royal Institute of Technology, SE-106 91 Stockholm, Sweden

[§] Institute of Molecular Science (ICMol), University of Valencia, 46980 Paterna, Spain

¥ School of Engineering, University of Warwick, Coventry, CV4 7AL, UK

Corresponding Authors

*bejan@kth.se (B.H.); toprak@kth.se (M.S.T.)

Structural and Microstructural Characterization

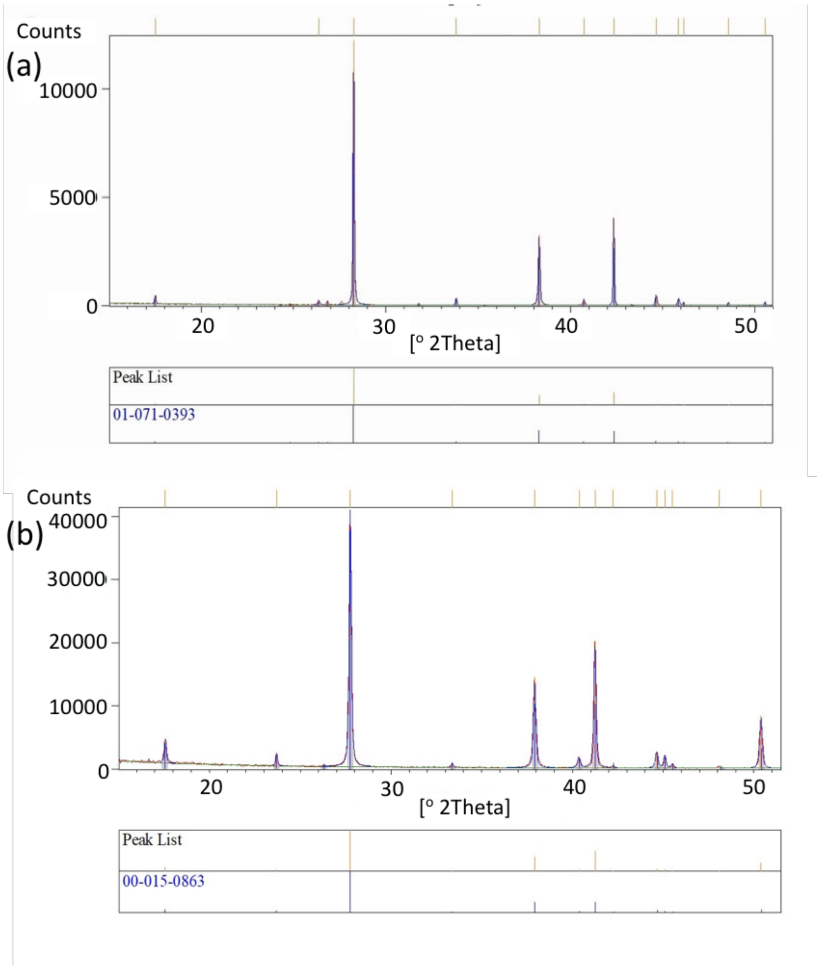

Figure S1. Powder XRD patterns of the as-made (a) Sb_2Te_3 and (b) Bi_2Te_3 nanoparticles through MW-assisted thermolysis route.

The phase structures are investigated by X-ray powder diffraction (XRPD) to identify the crystal structure, crystallinity, and the lattice parameters and compare the purity of the synthesized materials. XRPD analysis has been performed by using a Philips PANalytical X'Pert Pro Powder Diffractometer (Malvern Panalytical Ltd., Malvern, UK) with Cu-Kα radiation (λ = 1.54059 Å). A scan speed of 0.04 Å /s was used in continuous scan with a rotating sample holder. The crystalline phases are indexed to Bi_2Te_3 (ICDD: 00-015-0863) and Sb_2Te_3 (ICDD: 01-071-0393) with rhombohedral crystal structure, and the corresponding Bragg diffractions in comparison to the reference patterns are presented in Figure S1.

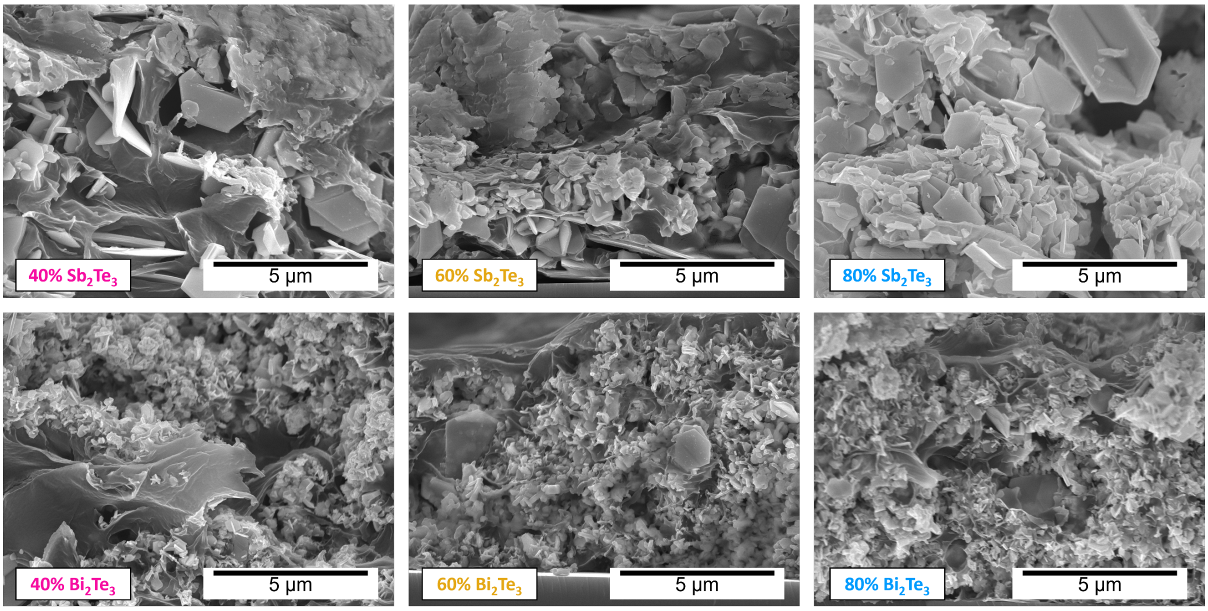

Figure S2. Cross-sectional SEM micrographs of some selected hybrid films with different content of Sb_2Te_3 and Bi_2Te_3 nanoparticles in the PMMA matrix. The film compositions are specified on each sub-figure

SEM micrographs of Bi_2Te_3 and Sb_2Te_3 samples at different magnifications are presented in **Figure S3**. All samples show particles with clear hexagonal, or truncated-edge hexagonal, platelet morphology, in agreement with their rhombohedral layered crystal structure, with slight change of platelet size from one sample to another. Sb_2Te_3 sample display larger lateral size as compared to Bi_2Te_3. Platelet size were evaluated using ImageJ software, using micrographs with different fields of view. Sb_2Te_3 exhibited average lateral platelet size about 1.5 μm (with std. dev. of 0.7 μm), and Bi_2Te_3 exhibited average particle size about 200 nm (with std. dev. of 95 nm). In both the cases the thickness of the platelets is around 50-70 nm. Crystallite size was estimated from the XRD data, using Williamson-Hall plot, in the range 160-600 nm, with a weighted average of about 200 nm for Sb_2Te_3 , while it is in the range of 50-100 nm with a weighted average of 70 nm for Bi_2Te_3. Due to the anisotropy in the particle morphology the crystallites are also expected to exhibit some anisotropy, the obtained size being viable in the lateral plane.

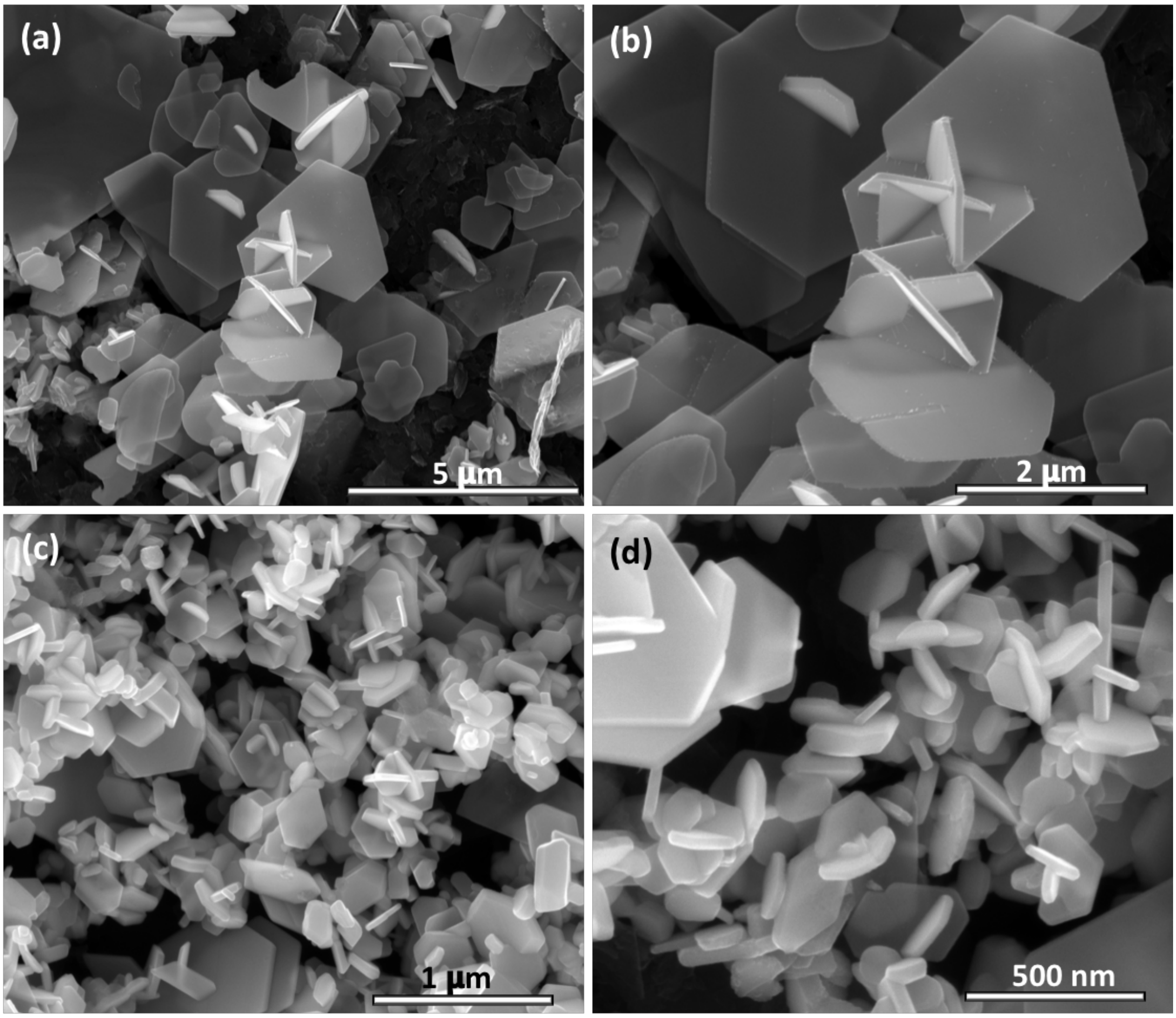

Figure S3. SEM micrographs of the as-made Sb_2Te_3 (a,b) and Bi_2Te_3 (c,d) nanoparticles, synthesized through MW-assisted thermolysis route, at different magnifications.

Percolation Theory

As the system shows significant value of a percolation threshold, we are inclined to consider that direction/model. The critical exponent in a percolation system is assumed to depend only on the dimensionality of the lattice and is independent of the details of the lattice structure.[1] Namely, for 3D systems the critical exponent acquires values between 1.6 and 2.0 and, for 2D systems, the critical exponent varies between 1.1 and 1.3.[2] However, in conducting systems the critical exponent does not follow the universal trend.[3] In systems based on insulating matrices with embedded conductive fillers and where the conduction process is controlled by interfiller tunneling, the inverted Swiss-cheese model can be applied, which predicts values of the critical exponent between 0.8 and 1.[2] An example is found in a work published by Wang et al.[4] in which

carbon nanotubes were introduced into a transparent PVDF matrix. In his case, the critical exponent has a value of 0.85, similar to that obtained by us when we use Bi_2Te_3 nanoparticles. The system can be considered 2D since the ratio between the thickness and the width of the film is very small. After the new measurements of electrical conductivity using the 4-point technique, the percolation curve settings have been updated in Figure 4 and the values of the critical exponent are close to 1.

When the percolation threshold of Sb_2Te_3 and Bi_2Te_3 hybrid films with the DDT linker are compared, the values obtained for Sb_2Te_3 are lower than that of Bi_2Te_3. As the percolation threshold depends on the particle size, the results suggest that Bi_2Te_3 nanoparticles are smaller than Sb_2Te_3. This was confirmed by the SEM micrographs of as-made Sb_2Te_3 and Bi_2Te_3 nanoparticles presented in **Figure S3**. In order to demonstrate the viability of this suggested outcome, we chose Sb_2Te_3-PMMA system for control experiments. We synthesized smaller Sb_2Te_3 platelets, by using the same synthetic process and only lowering the concentration of the precursors to half. DLS measurements are performed on the suspensions of previous and new batch of Sb_2Te_3 nanoparticles in isopropanol. Results are presented in **Figure S4a**, where the peak average dispersed size (by approximation to the volume of a sphere) of smaller sample is about 200 nm lower than the large ones used for the percolation study. Thereafter, a hybrid film is developed by using this and adapting the best performing film composition with 60% Sb_2Te_3 in the PMMA matrix, without the addition of DDT. The hybrid film with larger platelets showed a resistance of 600 Ω, while the films with smaller platelets showed about 600 kΩ (**Figure S4b**), which is about three orders of magnitude higher. This finding confirms the predictions

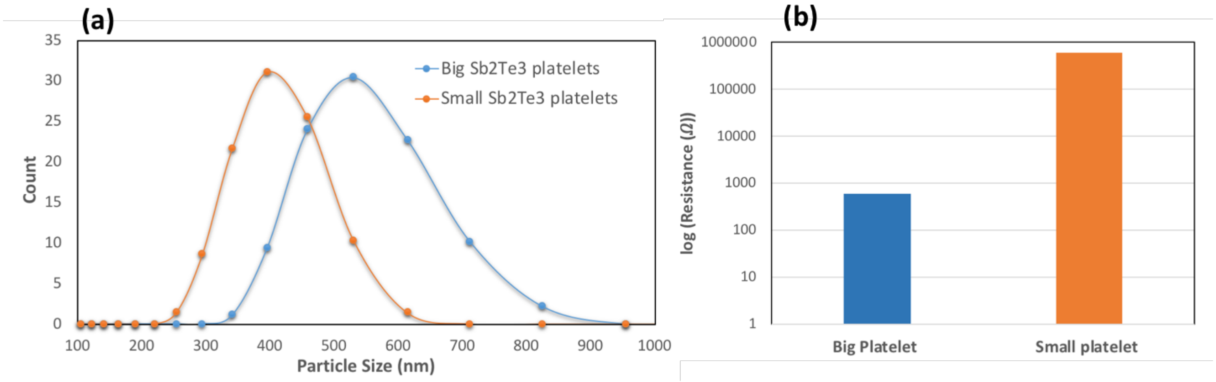

Figure S4. (a) Solvodynamic size distribution of Sb_2Te_3 platelets suspended in isopropanol, and (b) Resistance (in log scale) of the hybrid films (containing 60% Sb_2Te_3) prepared by dipsersing the small and large Sb_2Te_3 platelets in the PMMA matrix.

by the percolation theory, which allows the design of platelets of various lateral size to systematically study this correlation as a further research work.

Flexibility Tests

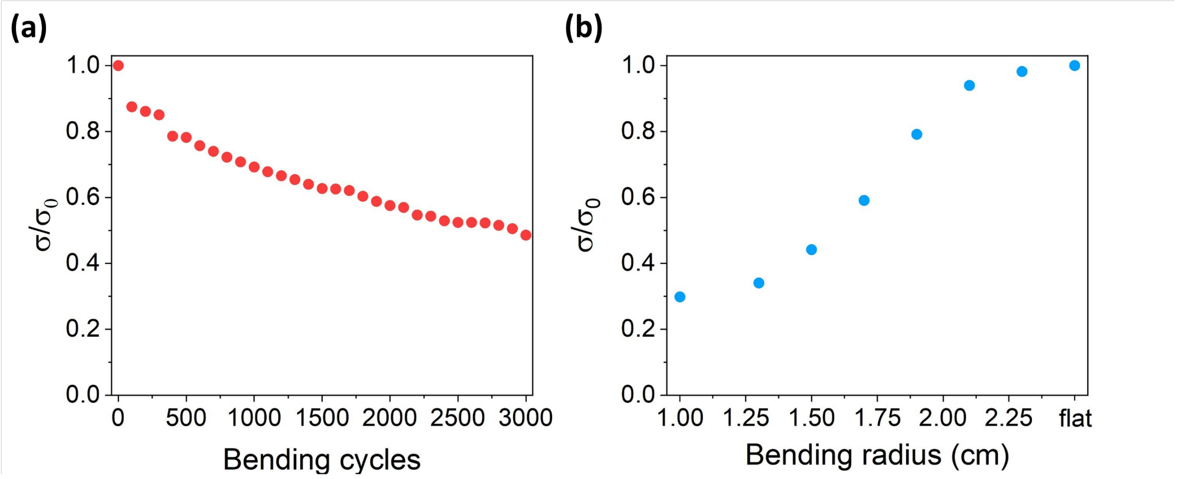

Figure S5. Flexibility test as a function of a) bending cycles and b) bending radius.

As a proof of concept, two flexibility tests have been carried out on Sb_2Te_3 –PMMA hybrid film with 60% Sb_2Te_3 content and DDT linker. For this, the deposition of the ink has been applied to a flexible PET substrate. **Figure S5a** shows the results of (2.5 cm long hybrid film on PET substrate) bending the film 3000 times on a 2 cm diameter cylinder and measuring the change in electrical conductivity after every 100 bendings. The conductivity of the film gradually decreases with the number of flexes until reaching a loss in electrical conductivity of 50% after 3000 flexes. On the other hand, **Figure S5b** shows the variation of electrical conductivity as a function of the bending radius. In this case, the electrical conductivity also decreases as the bending radius decreases and a loss of electrical conductivity by 70% is reached when the bending radius is as small as 1 cm. These results clearly indicate that formulated hybrid films based on Sb_2Te_3 (and Bi_2Te_3) nanoparticles with DDT and in the PMMA matrix are not particularly flexible. However, we must remember that a film composed solely of Sb_2Te_3 (or Bi_2Te_3) nanoparticles deposited on a flexible substrate such as PET would almost completely lose electrical conductivity after a few bending cycles, since in this case there would be no glue effect of the polymeric matrix. In addition, we must remember that the polymeric matrix, PMMA, used in this work is a matrix that is rigid at room temperature since its glass transition temperature, T_g, is around 110 ºC.[5] Therefore, the developed hybrid-films will only have a

flexible behavior when the working temperature is higher than 110 ºC since it is at this temperature when the polymeric matrix begins to be more fluid and, therefore, can be handled without breaking. With the choice of other flexible polymers as the matrix a higher flexibility could be achieved, with much less degradation of the transport performance.

Transport Property Evaluation

The electronic transport properties of the hybrid films were determined by the measurement of electrical conductivity (***σ***) and the Seebeck coefficient (***S***). The ***σ*** was determined by the Van der Pauw equation (Eq. S1), inserting four equidistant contacts of conductive silver paint on the surface of the films (**Figure S6**). Next, a current was applied between two points and the potential between the other two points was measured, obtaining R_1. To obtain R_2, we applied a current intensity between two other points and the potential between the remaining two was measured. Knowing the values of R_1, R_2 and the thickness (d), the electrical resistivity (ρ) of the film was obtained, which was then converted to electrical conductivity.

$$e^{-R_1\frac{\pi.d}{\rho}} + e^{-R_2\frac{\pi.d}{\rho}} = 1 \qquad \text{(Eq. S1)}$$

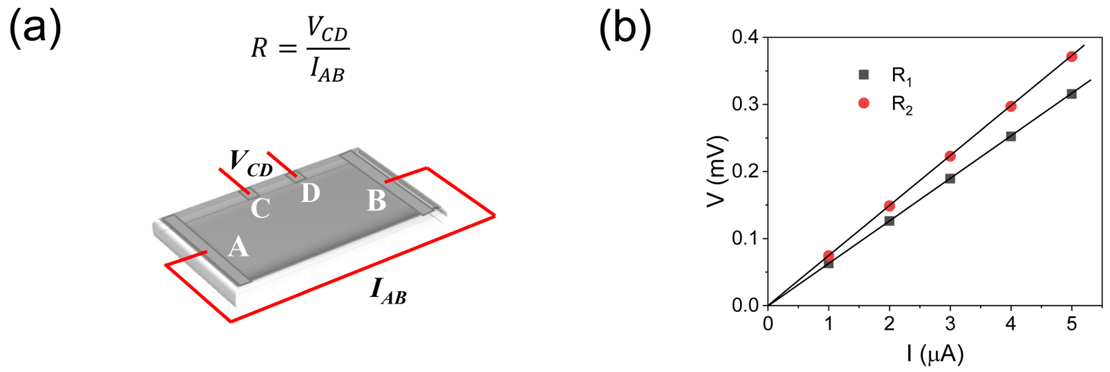

Figure S6. (a) Contacts configurations to measure electrical resistance R_1 and R_2. (b) Voltage as a function of the current to determine R_1 and R_2.

The ***S*** was determined at room temperature using a homemade system consisting of two copper blocks (**Figure S7**). One of the copper blocks was heated by a Peltier module while the other was kept at room temperature. The temperature values at the hot and cold end of the

samples were recorded using two K-type thermocouples connected to PicoLog software. The hot-side temperature was gradually increased to reach a gradient of 60 °C by applying a voltage to the Peltier module with a source supplier (Keithley 2280S). The ***S*** potential generated was recorded with a Keithley 2450 source meter. By plotting the ***S*** voltage generated as a function of the temperature gradient, a linear distribution was obtained, where slope is the ***S***.

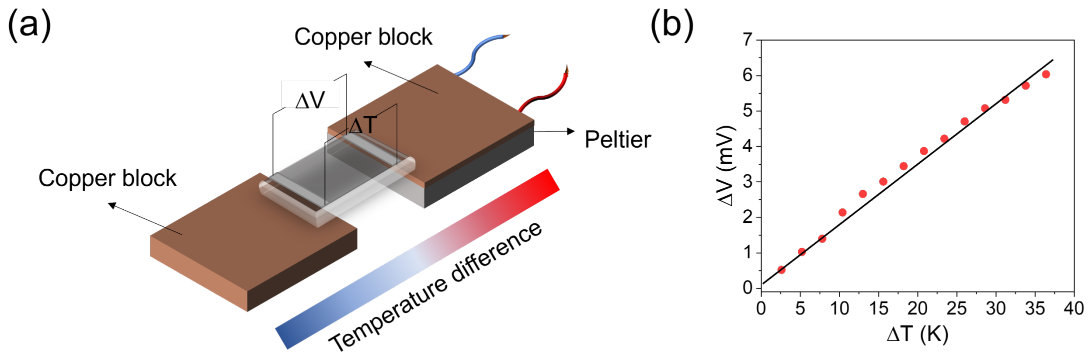

Figure S7. (a) Scheme of the home-made set up to measure the Seebeck coefficient. (b) Voltage generated as a function of the temperature difference, where the sloop is the Seebeck coefficient.

In order to optimize the thermoelectric films, the influence of the film thickness on the electrical conductivity was studied by varying the number of layers deposited on the substrate. **Figure S8** represents the study carried out previously to the percolation curve for the hybrid films containing 60wt% Sb_2Te_3 or Bi_2Te_3 nanoparticles. It was observed that for both the Sb_2Te_3 (**Figure S8a**) Bi_2Te_3 (**Figure S8b**) films an optimal value of electrical conductivity is found with the film thickness around 3.5 μm, which corresponds to the thickness after 4-layer deposition (The thickness of the films was measured by using a profilometer (Profiler KLA-Tencor P15, CA, USA), while the lateral dimension was measured using a caliper). For this reason, during the development of the work it was decided to use 4 layers in all the prepared films.

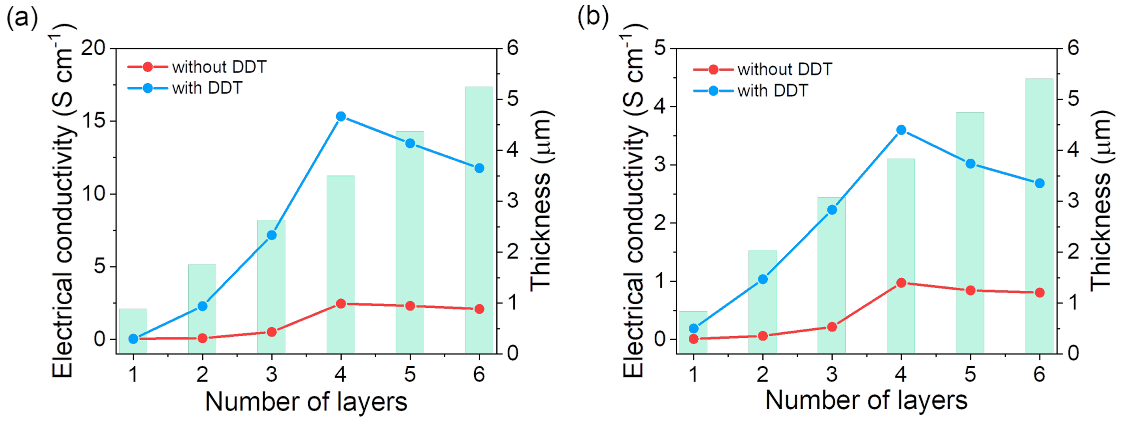

Figure S8. Electrical conductivity and film thickness as a function of the number of layers of the hybrid films with and without DDT for: (a) 60% Sb_2Te_3 - PMMA, and (b) 60% Bi_2Te_3 - PMMA.

References

(1) Sahimi, M.; Hughes, B. D.; Scriven, L. E.; Davis, H. T. Critical Exponent of Percolation Conductivity by Finite-Size Scaling. *J. Phys. C Solid State Phys.* **1983**, *16* (16), L521--L527. https://doi.org/10.1088/0022-3719/16/16/004.

(2) Nan, C.-W. Physics of Inhomogeneous Inorganic Materials. *Prog. Mater. Sci.* **1993**, *37* (1), 1–116. https://doi.org/https://doi.org/10.1016/0079-6425(93)90004-5.

(3) Yoon, S.; Lee, S.-I. Possible Breakdown of the Universality of the Conductivity Critical Exponent in an Anisotropic Percolation System. *Phys. B Condens. Matter* **1990**, *167* (2), 133–137. https://doi.org/https://doi.org/10.1016/0921-4526(90)90005-F.

(4) Wang, L.; Dang, Z.-M. Carbon Nanotube Composites with High Dielectric Constant at Low Percolation Threshold. *Appl. Phys. Lett.* **2005**, *87* (4), 42903. https://doi.org/10.1063/1.1996842.

(5) Roth, C. B.; Pound, A.; Kamp, S. W.; Murray, C. A.; Dutcher, J. R. Molecular-Weight Dependence of the Glass Transition Temperature of Freely-Standing Poly(Methyl Methacrylate) Films. *Eur. Phys. J. E* **2006**, *20* (4), 441–448. https://doi.org/10.1140/epje/i2006-10034-0.
